# Micro-fragmented adipose tissue (mFAT) associated with arthroscopic debridement provides functional improvement in knee osteoarthritis: a randomized controlled trial

**DOI:** 10.1007/s00167-022-07101-4

**Published:** 2022-08-30

**Authors:** Michele Ulivi, Valentina Meroni, Marco Viganò, Alessandra Colombini, Michele D. M. Lombardo, Nicolò Rossi, Luca Orlandini, Carmelo Messina, Luca M. Sconfienza, Giuseppe M. Peretti, Laura Mangiavini, Laura de Girolamo

**Affiliations:** 1 IRCCS Istituto Ortopedico Galeazzi, via Riccardo Galeazzi 4, 20161 Milan, Italy; 2 Residency Program in Orthopedics and Traumatology, University of Milan, via Festa del Perdono 7, 20122 Milan, Italy; 3 Department of Biomedical Sciences for Health, University of Milan, Via Luigi Mangiagalli, 31, 20133 Milan, Italy

**Keywords:** Knee osteoarthritis, Arthroscopic debridement, Micro-fragmented adipose tissue, Regenerative medicine, Cartilage biomarkers

## Abstract

**Purpose:**

Current conservative treatments for knee OA provide limited benefits, with symptoms relief for a short amount of time. Regenerative medicine approaches such as the use of microfragmented adipose tissue (mFAT) showed promising results in terms of durable effects and the possibility to enhance tissue healing and counteract the progression of the pathology. Nevertheless, up to today, the large part of clinical data about mFAT use refers to uncontrolled studies, especially in the surgical setting. The purpose of this study was to evaluate the effectiveness of mFAT applied in association with arthroscopic debridement (AD) for the treatment of knee OA, in terms of symptoms relief and tissue healing.

**Methods:**

This study is a prospective, randomized controlled clinical trial. 78 patients affected by knee OA grade 3–4 according to KL classification were randomly assigned to AD or AD + mFAT treatment groups. Clinical, radiological and serological assessments were performed at 6 months after treatment. Additional clinical evaluation was performed at the end of the study with an average follow-up of 26.1 ± 9.5 months. VAS, KOOS, WOMAC and SF-12 were also collected at both timepoints, KSS only at 6 months.

**Results:**

Treatment with AD + mFAT improved functional scores at both 6 months (KOOS-PS: + 11.7 ± 20.2 vs + 24.4 ± 22.5, in AD and AD + mFAT, respectively, *p* = 0.024; KSS: + 14.9 ± 15.9 vs + 24.8 ± 23.5, in AD and AD + mFAT, respectively, *p* = 0.046) and 24-month follow-ups (KOOS-PS Functional subscale: − 2.0 ± 3.5 vs − 4.7 ± 4.2, in AD and AD + mFAT, respectively, *p* = 0.012). Lower T2-mapping scores were obtained in AD + mFAT-treated group in medial and lateral condyle compartments (*p* < 0.001). Slight increase was observed in the levels of a serum biomarker of cartilage deposition (PIIINP) in both groups at 6-month follow-up (*p* = 0.037).

**Conclusion:**

mFAT improves functional outcome and MRI appearance when used in association with AD, therefore supporting its use in the treatment of knee OA in an arthroscopic setting.

**Supplementary Information:**

The online version contains supplementary material available at 10.1007/s00167-022-07101-4.

## Introduction

Knee osteoarthritis (OA) is a common condition, and a variety of conservative solutions has been proposed to control the related symptomatology. Traditional conservative therapies, including but not limited to anti-inflammatory drugs and viscosupplementation, showed short-term benefits for the management of symptoms, but they have no effect on pathology progression or tissue restoration. [10]. In this scenario, an increasing attention has been addressed to the development of treatments potentially targeting the degenerative processes underlying the pathology, including mesenchymal stromal cell (MSC)-based therapy [22, 40]. As an alternative to the use of in vitro expanded MSCs, that require extensive cell manipulation that make them advanced-therapy medicinal products (ATMPs), one step approaches based on MSCs/pericytes-derived products have been considered as promising for the treatment of knee OA [20, 25, 32]. Adipose tissue, in particular, represents an easy accessible source of MSCs and its micro-fragmentation (microfat or mFAT) allows to quickly harvest a relevant volume of a minimally manipulated tissue composed by clusters containing MSCs [38]. This procedure empowers tissue regeneration by improving MSCs secretion of cytokines and angiogenic factors [7, 20, 25, 32, 41]. Moreover, mFAT contains an inferior amount of leukocytes [3] and supra-adventitial-adipose stromal cells with respect to raw adipose tissue, along with an enrichment in endothelial progenitors [31], that have been described to sustain proliferation and differentiation in an interplay with tissue resident cells [17, 19]. The biological composition of mFAT suggests the ability of micro-fragmentation technology to reduce the presence of pro-inflammatory elements, while promoting the interaction between endothelial progenitors and MSCs/pericytes, in turn activating their anti-inflammatory and pro-regenerative potential [31]. Several clinical studies investigating the intra-articular injection of mFAT for the treatment of knee OA showed significant pain and articular stiffness reduction, along with an increased articular mobility [26], together with improvements in cartilage quality as assessed by dGEMRIC (delayed gadolinium enhanced MRI of cartilage) index at 12- and 24-month follow-up [4, 11, 12]. Cohort studies confirmed these results as well as the safety of the treatment [2, 23, 24, 29]. When used as surgical adjuvant in different surgical settings, including anterior cruciate ligament (ACL) and lateral collateral ligament (LCL) reconstruction, meniscectomy, osteotomy and debridement, mFAT demonstrated to be a safe and effective approach [33, 34]. Nevertheless, the lack of a control group in most studies did not allow drawing definitive conclusion up to today. In this scenario, RCTs are needed to reinforce these observations and shed light on the real potential of mFAT in the treatment of OA.

This work reports the results of a randomized controlled trial (RCT) comparing the efficacy of autologous mFAT (obtained using Lipogems^®^, Lipogems International SpA, Milan, Italy) in association with arthroscopic debridement (AD) with respect to arthroscopic debridement alone for the treatment of severe knee OA (Kellgren–Lawrence, KL 3–4). While arthroscopic debridement does not represent an effective treatment for severe knee OA [28], it has been selected as a convenient surgical control for the present study to standardize the joint basal condition in each patient.

The study hypothesis is that mFAT would improve the clinical outcomes in comparison to AD procedure alone.

## Methods

### Study design

The study is a single-center, interventional, prospective, randomized, controlled study. Patients were enrolled after approval of the study protocol by the relevant Ethics Committee (Approval number 150/INT/2016 released on 2017 March the 9th). The protocol complies with the current Declaration of Helsinki, the EN ISO 14155: 1 and EN ISO 141155: 22 standards and the Good Clinical Practice (GCP).

Subjects, homogeneous for sex, age and physical activity, were randomly assigned to two treatment groups (Arthroscopic Debridement or Arthroscopic Debridement + mFAT) using a computer-generated 1:1 table.

A total of 78 patients with knee osteoarthritis were divided into 2 groups: Group I (Arthroscopic Debridement, AD) consisting of 39 patients undergoing arthroscopic debridement alone and Group II (AD + mFAT) consisting of 39 patients undergoing arthroscopic debridement and concomitant injection of autologous microfragmented lipoaspirated tissue (Lipogems^®^) [30].

### Patient selection

The study was proposed to any patient affected by knee OA (3–4 KL) who met the inclusion/exclusion criteria and came to the Galeazzi Orthopaedic Institute between 13/06/2017 and 04/12/2019. Inclusion criteria were: age range 45–75 years, knee osteoarthritis grade 3–4 according to Kellgren-Lawrence classification [37], Visual Analog Scale (VAS) joint pain ≥ 6 at the time of enrollment, ligament instability ≤ grade II, mechanical axis with axial deviation (varus or valgus) under load not exceeding 10°, normal coagulation parameters (PT (INR) < 1.5) and BMI between 18 and 30 kg/m^2^.

Exclusion criteria included: previous treatment for traumatic injuries (tibial osteotomies, tibial plateau fractures), viscosupplementation, injection of cortisone or meniscal surgery within 3 months of the procedure, vascular necrosis/osteonecrosis of the knee previous articular infections. Other exclusion criteria were presence of autoimmune diseases, general infections within the previous 6 weeks, diabetes, malignant neoplasms, metabolic diseases, cardiac, pulmonary, neurological pathologies, bleeding disorders, pregnancy. Patients undergoing radiation therapy, chemotherapy, immunosuppression, steroid therapy within the previous 8 weeks were also excluded as well as drug and/or alcoholic abuser and heavy smokers.

### Surgical procedures

The arthroscopic knee debridement was performed by two surgeons involved in the study. The procedure consists of abundant joint lavage aimed at removing joint debris, unstable chondral fragments, cartilaginous mobile bodies, osteophytes, parts of degenerated menisci and hypertrophic synovial membrane. Other procedures such as microfractures, massive meniscectomies or synovectomies not performed.

The adipose tissue was harvested by lipoaspiration from the abdominal or thigh subcutaneous adipose tissue, depending on the physical characteristics of the patient, during the same operating session by a plastic surgeon. The patients were anesthetized locally in the harvest area (250 ml of physiological solution + 2 ml marcaine vials + 1 ml adrenaline vial) using a spinal needle, diffusely in the subcutaneous layer. The adipose tissue was harvested through a 13G cannula, equipped with a tip specifically designed not to damage the tissue, and connected to a syringe to dose the suction pressure to reduce the traumatic action on the cells. Approximately 100–120 ml of adipose tissue were collected from each patient. The adipose tissue obtained was then processed using the 120 ml Lipogems^®^ device (Lipogems International SpA, Milan, Italy), a closed, full-immersion, low-pressure cylindrical system, aimed to wash and resize the adipose tissue [3]. Briefly, the lipoaspirate was injected through a large filter placed on top of the cylinder that contained stainless steel beads and had been prefilled with saline before beginning the processing to avoid cell damage. When the whole amount of lipoaspirate tissue was transferred to the device, the cylinder was quickly shaken for about one minute to emulsify residues of lipid droplets which were subsequently removed together with contaminating blood components by a gravity counterflow of saline solution. The washed and resized adipose tissue migrated to the top of the Lipogems device and was collected by passing through a size reduction filter into 10-ml syringes connected to the upper opening of the device. On average, per each patient 1 syringe of approximately 10 ml of mFAT was obtained. At the end of the arthroscopic debridement, 6–8 ml of mFAT obtained was injected by an 18-gauge syringe.

The patients were advised to follow a simple rehabilitation protocol including exercises for flexion–extension recovery and quadriceps strengthening, while starting to walk with two crutches with partial weight bearing. Concerning the post-operative use of drugs, patients were prescribed Ibuprofen 600 mg two times/day and, if further needed, Paracetamol 1 g (max three times/day); Clexane 4000 UI for the first 12 days or until complete recovery of deambulation and, only for Lipogems-group patients, Cefixime 400 mg 1 cp for 5 days.

### Clinical–functional and imaging post-operative evaluation protocol

The patients in both study groups were evaluated using an accurate clinical–functional and imaging post-operative evaluation protocol.

A clinical visit was performed before (T0) and 6 months after the treatment (T6). At the same time point, VAS [15], Knee injury and Osteoarthritis Outcome Score (KOOS)-PS (and its subscales activity (A), and function (F)) [8], Knee Society Score (KSS) (and the subscale KSS-F) [13], Western Ontario and McMaster Universities osteoarthritis index (WOMAC) [35], short form-12 (SF-12, physical and mental component subscales, PCS/MCS) [16] were also collected. In particular, higher values of KOOS-PS, SF-12 and KSS are representative of better clinical conditions; on the contrary, lower values of VAS, WOMAC and KOOS-PS subscales (Activity and Function) are representative of better clinical conditions.

At longer follow-up (13–42 months), the patients were reached out by phone and asked to fill the same patient-reported outcome measures (PROMs: VAS, KOOS, WOMAC, SF-12).

### Radiological analysis

All the evaluations were performed by a senior radiologist who was blinded to the group allocation of the patients. All patients underwent routine pre-operative X-ray and magnetic resonance imaging evaluation (MRI) for disease assessment (T0), together with a 6-month MRI follow-up (T6). All the follow-up MRI examinations were executed on a 1.5-T MRI scanner (Avanto, Siemens Medical Solution, Erlangen, Germany), using: sagittal turbo spin-echo (TSE) T1-weighted sequence (time of repetition, TR = 600–610 ms, time of echo, TE = 8–15 ms), TSE T2-weighted (TR = 3900–5500 ms, TE = 80–110 ms) on the axial and coronal planes, and fat-saturated proton density sequences on the three planes (TR = 2800–3100 ms, TE = 30–40 ms). Field of view was 180 mm for the axial and coronal images and 160 mm for the sagittal images. The radiological assessments considered the femorotibial angle, % Weight-Bearing Line (WBL) and Outerbridge classification.

### T2 mapping analysis

For T2 mapping, a sagittal multiecho sequence (TR = 3500 ms, TE = 100 ms, number of echoes = 10, matrix 384 × 384 pixels, acquisition voxel 0.7 × 0.7 × 4 mm, bandwidth 180 Hz; slice thickness 4 mm, slice gap 4.4 mm), with a field of view of 160 mm was added. MR images were evaluated by a senior musculoskeletal radiologist who manually drew four regions of interest (ROIs), to include the whole thickness of the cartilage of medial and lateral side, both at the level of femur condyles and tibia. As for previous studies [6], the analysis was performed using a dedicated software (Olea Sphere^®^ 3.0 software, Olea Medical^®^, La Ciotat, France). Before the analysis, the software pre-processed all images with an automatic correction to remove possible motion artefacts.

### Biochemical analysis

The blood samples were collected pre-operatively (T0) and at 6-month (T6) follow-up, allowing them to clot at room temperature before centrifugation (3000*g*, 10 min). The sera were aliquoted and stored at − 80 °C until assayed. Cross-linked C-Telopeptide of Type II collagen (CTx-II, Cusabio Technologies, Houston, Texas, USA) and N-Terminal Procollagen III Propeptide (PIIINP, Elabscience, Houston, Texas, USA) enzyme-linked immunosorbent assays (ELISA) kits were used to determine the concentration of CTx-II and PIIINP following the manufacturer’s indications. The range of detection for CTX-II was 312–20,000 pg/ml, sensitivity of 78.0 pg/ml, whereas for PIIINP it was 23.4–1500 pg/ml with a sensitivity of 14.1 pg/ml.

### Sample size and statistical analysis

The sample size was calculated using KOOS-PS as the primary outcome. A difference of ten points at 6 months between the two different groups of patients was assumed (*α* = 0.05; *β* = 0.20). Considering an intra-group standard deviation of 15 points, 70 subjects were necessary to obtain the desired statistical power (35 per group). Considering a 10% dropout rate, 8 patients were added to this number, for a total of 78 subjects (39 per group).

Analyses were performed using R software v4.0.3 (R Core Team, Wien, Austria). Continuous variables were tested for normal distribution using Shapiro–Wilk test. According to the result of the test, between groups comparisons were performed with unpaired *t* test or Mann–Whitney test, while paired tests were used to compare baseline and follow-up results in the whole cohort. Changes in clinical scores were measured using the difference between follow-up and baseline values. Percentage change was calculated as the ratio between follow-up—baseline absolute change and baseline value * 100. Between groups differences in the categorical variables were tested using Fisher’s exact test. Multiple regression linear models were used to assess the relation of CTX-II and PIIINP serum levels and the clinical scores after adjustment for age and gender. The contemporary influence of two variables were assessed by a two-way ANOVA test with Bonferroni post hoc test. *P* values < 0.05 were considered statistically significant.

## Results

### Patients’ demographics

**Fig. 1:**
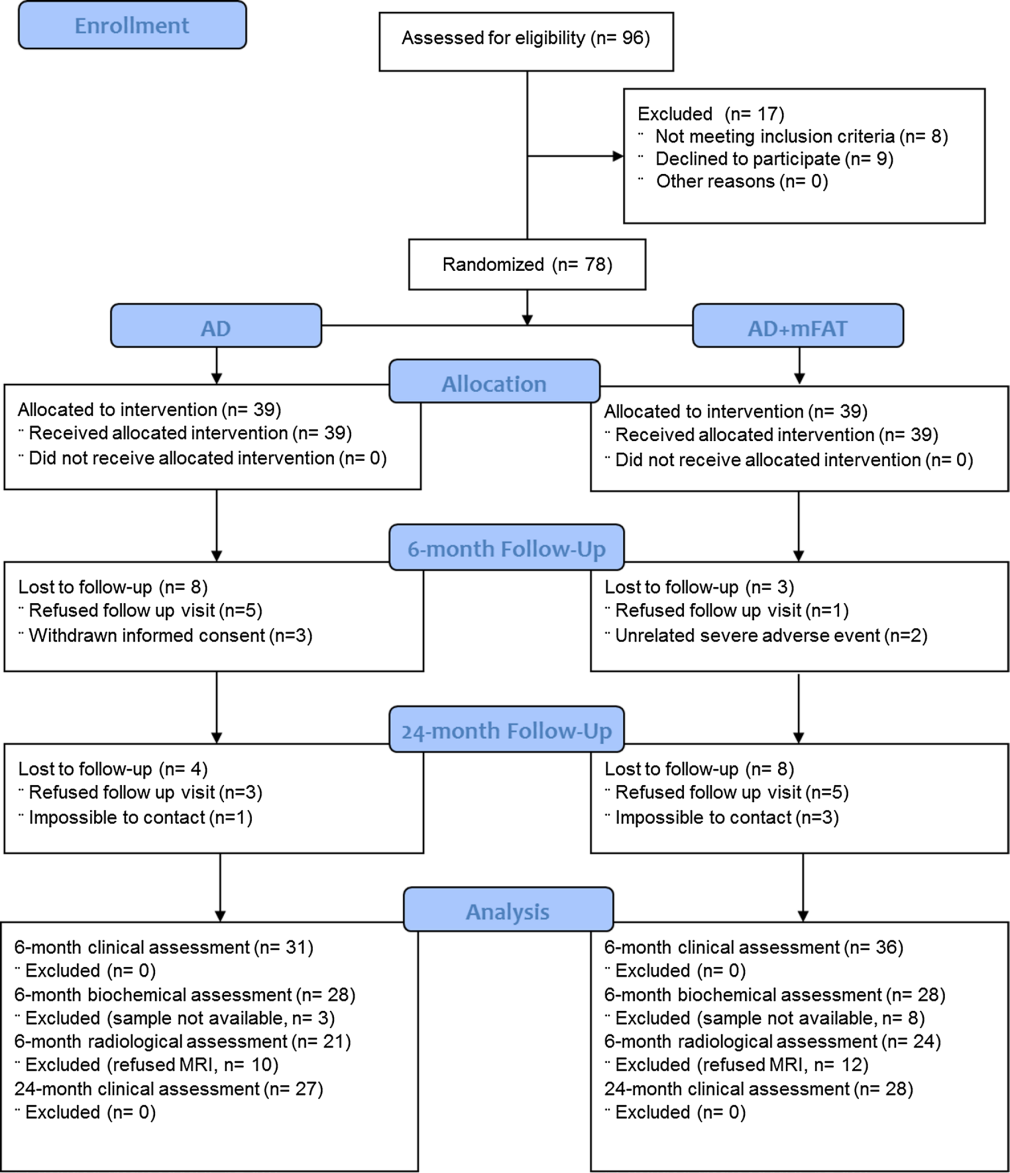
PRISMA flowchart. Flowchart of patients’ enrollment, treatment, follow-up and analysis

Seventy-eight patients were enrolled in the study (34 females, 44 males), with a mean age of 60.7 ± 7.9 years old. Twelve (12) patients did not complete the 6-month follow-up, with no difference between study groups (AD 9; AD + mFAT 3; *p* = 0.114) (Fig. 1) No significant differences in terms of baseline PROMs were observed between patients who eventually dropout and patients who completed the study. The final analysis was conducted on 67 patients.

Patients in the two groups had similar age and gender distribution.

At baseline, the two groups showed similar values for what concern VAS, WOMAC, KSS-F, SF-12 MCS and SF-12 PCS, while the groups differ significantly considering KSS (*p* < 0.001) and KOOS-PS (*p* < 0.05) (Supplementary Table 1).

### Safety of the treatment

Four Adverse Events (AEs), all in the AD + mFAT group, were reported and notified to the Ethics Committee as well as to the National Competent Authority. Three of them were classified as serious but not related to the procedure. In details, they included a case of breast cancer diagnosed 5 months after the procedure, a case of multiple contusions after an accidental fall 8 months after the procedure and a case of pulmonary embolism occurred three months after intervention and therefore, due to the time lap between intervention and event onset, considered as non-related to the procedure.

A mild procedure-related adverse event also occurred at the site of collection of the adipose tissue (hematoma formation), that resolved spontaneously in few days.

### Clinical evaluations

**Fig. 2:**
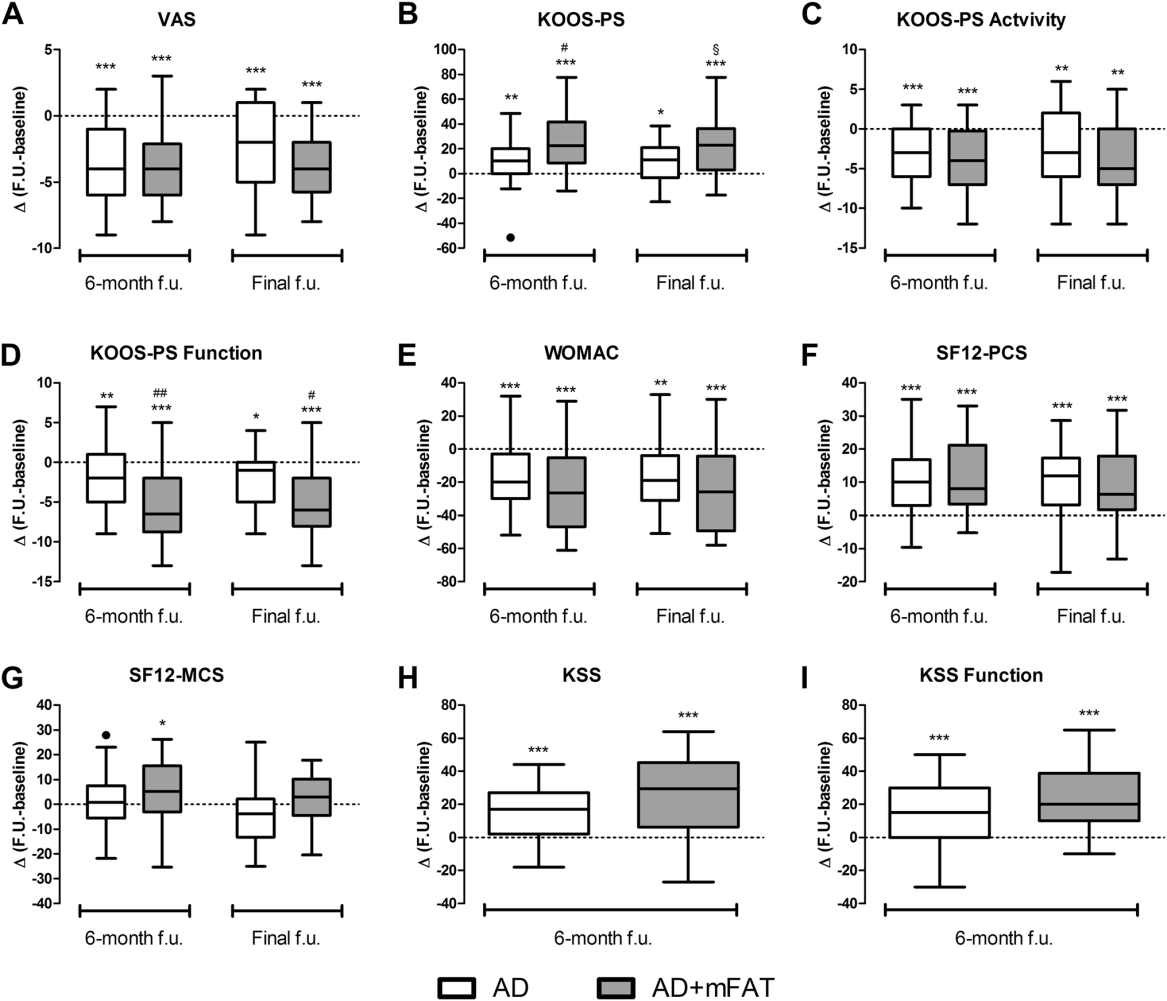
Changes in patients reported outcome measures. Changes at 6 and final follow-up (calculated as value at follow-up – value at baseline) for VAS (**A**), KOOS-PS (**B**), KOOS-PS Activity subscale (**C**), KOOS-PS Function subscale (**D**), WOMAC (**E**), SF-12 Physical Component Score (**F**) and SF-12 Mental Component Score (**G**). Concerning KSS (**H**) and its functional subscale (**I**) values were collected at baseline and 6-month follow-up only. *p < 0.05, **p < 0.01, ***p < 0.001 vs baseline (within-group difference); ^§^*p* < 0.1 (tendency), ^#^*p* < 0.05, ^##^*p* < 0.01 vs AD group (between-group difference). Clinical improvements are represented as decreases in VAS, KOOS-PS Activity subscale, KOOS-PS Function subscale and WOMAC, and as increases in KOOS-PS, KSS, KSS Function subscale, SF-12 MCS and SF-12 PCS

In general, higher improvements were observed in patients treated with AD + mFAT and AD alone for all scores (Fig. 2), even if at the 6-month follow-up, statistical significant differences between groups were observed only for what concern KOOS-PS and KSS. The differences in KOOS-PS was mainly due to the functional subscale (F), which showed a significant improvement (*p* = 0.005).

At the mean final follow-up of 26.1 ± 9.5 months, the PROMs of 55 patients (28 in the AD + mFAT group and 27 in the AD group) were collected. The mean follow-up was similar between the two groups (AD 25.6 ± 9.9 months; AD + mFAT 26.7 ± 9.3 months; *p* = 0.545).

Considering changes from baseline at the last follow-up, there are no significant differences between groups, although a tendency was observed in KOOS-PS improvements (*p* = 0.064) (Fig. 2). Again, the reduction in this parameter was influenced by functional subscale in particular (*p* = 0.012) (Fig. 2D).

Overall, the significant intra-group improvements observed at 6 months with respect to baseline were maintained in both groups at the final follow-up for all PROMs tested at the final follow-up (VAS, KOOS-PS, SF12-PCS, SF12-MCS, WOMAC) (Fig. 2). A multivariate analysis considering both the time of observation and the study group confirmed the significant increases in time for both groups in KOOS-PS, VAS, SF12-P and WOMAC (*p* < 0.001 each), as well as the significant influence of mFAT on KOOS-PS increment during time (group*time interaction: *p* = 0.006). Values for each scores and changes with respect to baseline are reported in Supplementary Table 1.

### Changes in serum levels of CTx-II and PIIINP

Data about the serum levels of CTx-II and PIIINP were available for 56 patients, 28 in control group and 28 in treatment group. The two groups showed different values at baseline for both markers, even if these differences were not statistically significant. A slight and non-significant reduction was observed in the whole cohort for what concern the catabolic marker CTx-II (*p* = 0.527). Also analyzing the two groups separately, no significant differences emerged between DA and AD + mFAT groups (*p* = 0.724). In the whole cohort, PIIINP showed a slight increase with respect to baseline, which resulted statistically significant (*p* = 0.037). Nevertheless, the median percentage increase was very small (7%). No differences were observed comparing AD and AD + mFAT. Table 1 summarizes these findings.

**Table 1:** CTX-II and PIIINP levels in patients’ serum before and after the procedures

	Overall (*n* = 56)			AD (*n* = 28)			AD + mFAT (*n* = 28)		
	Baseline	6 months	*δ*	Baseline	6 months	*δ*	Baseline	6 months	*δ*
CTX-II (pg/mL)	962 ± 881	882 ± 588	− 80 ± 521	805 ± 716	774 ± 535	− 89 ± 439	1119 ± 752	1047 ± 601	− 71 ± 600
PIIIPN (pg/mL)	1880 ± 1922	1957 ± 1814*	77 ± 499	2252 ± 2356	2261 ± 2165	8 ± 573	1508 ± 1299	1655 ± 1353	146 ± 412

*p < 0.05 vs baseline. Data expressed as mean ± standard deviation

### Relation between the clinical scores and the serum levels of CTX-II and PIIINP

Preoperative levels of serum CTX-II demonstrated a significant correlation with pre-operative KOOS-PS (Spearman’s *r* = − 0.342, *p* = 0.009), KSS score (Spearman’s *r* = − 0.364, *p* = 0.006) and KSS-F (Spearman’s *r* = − 0.385, *p* = 0.003). After adjustment for age and gender, elements that significantly influence these scores, only KSS and KSS-F maintain the significance (*p* = 0.014 and *p* = 0.018, respectively). Serum levels of PIIINP did not correlate with any score.

### Radiological evaluations

Patients in AD and AD + mFAT groups demonstrated similar Outerbridge classification before intervention in both medial and lateral tibio-femoral compartments (Table 2).

**Table 2:** Outerbridge classifications in medial and lateral tibio-femoral compartments

Compartment	AD^a^	AD + mFAT^a^	*P* value
Medial	Class 1: 1	Class 1: 4	0.889
	Class 2: 7	Class 2: 2	
	Class 3: 3	Class 3: 4	
	Class 4: 9	Class 4: 11	
Lateral	Class 1: 4	Class 1: 4	0.971
	Class 2: 9	Class 2: 11	
	Class 3: 6	Class 3: 4	
	Class 4: 1	Class 4: 2	

^a^X-rays were available for 20 and 21 patients in AD and AD + mFAT group, respectively

**Fig. 3:**
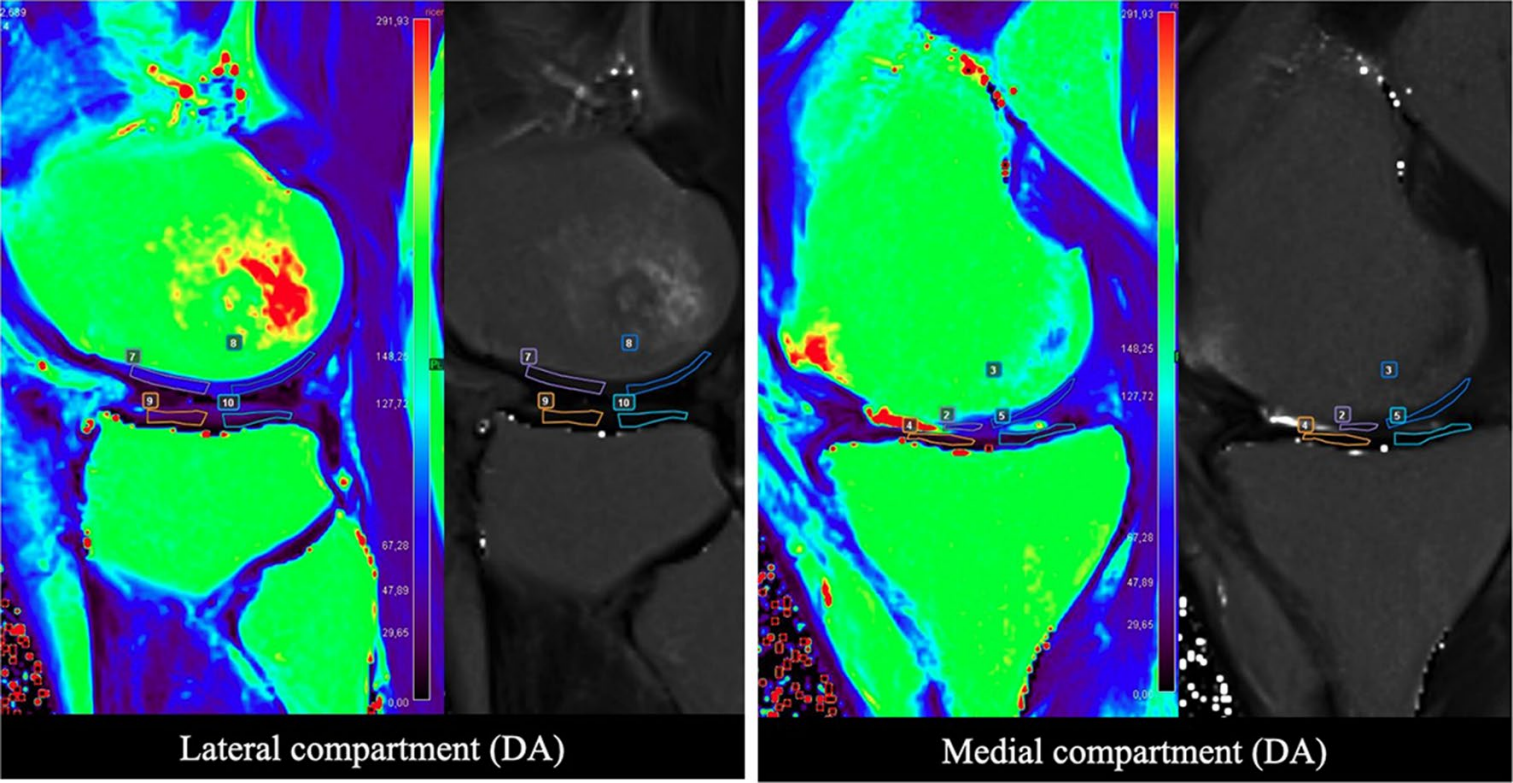
Representative MRI image for patients in DA group. T2 mapping evaluation in a 50-year-old male patient treated with AD and diagnosed with an MRI Outerbridge grade IV at tibio-femoral joint. Regions of interest positioning on both tibia and femur (ROIs) is showed on the lateral side of the knee (left image) and medial side (right image), with corresponding native mapping image used to properly locate the ROIs. T2-mapping values range from 0 to 291.93

**Fig. 4:**
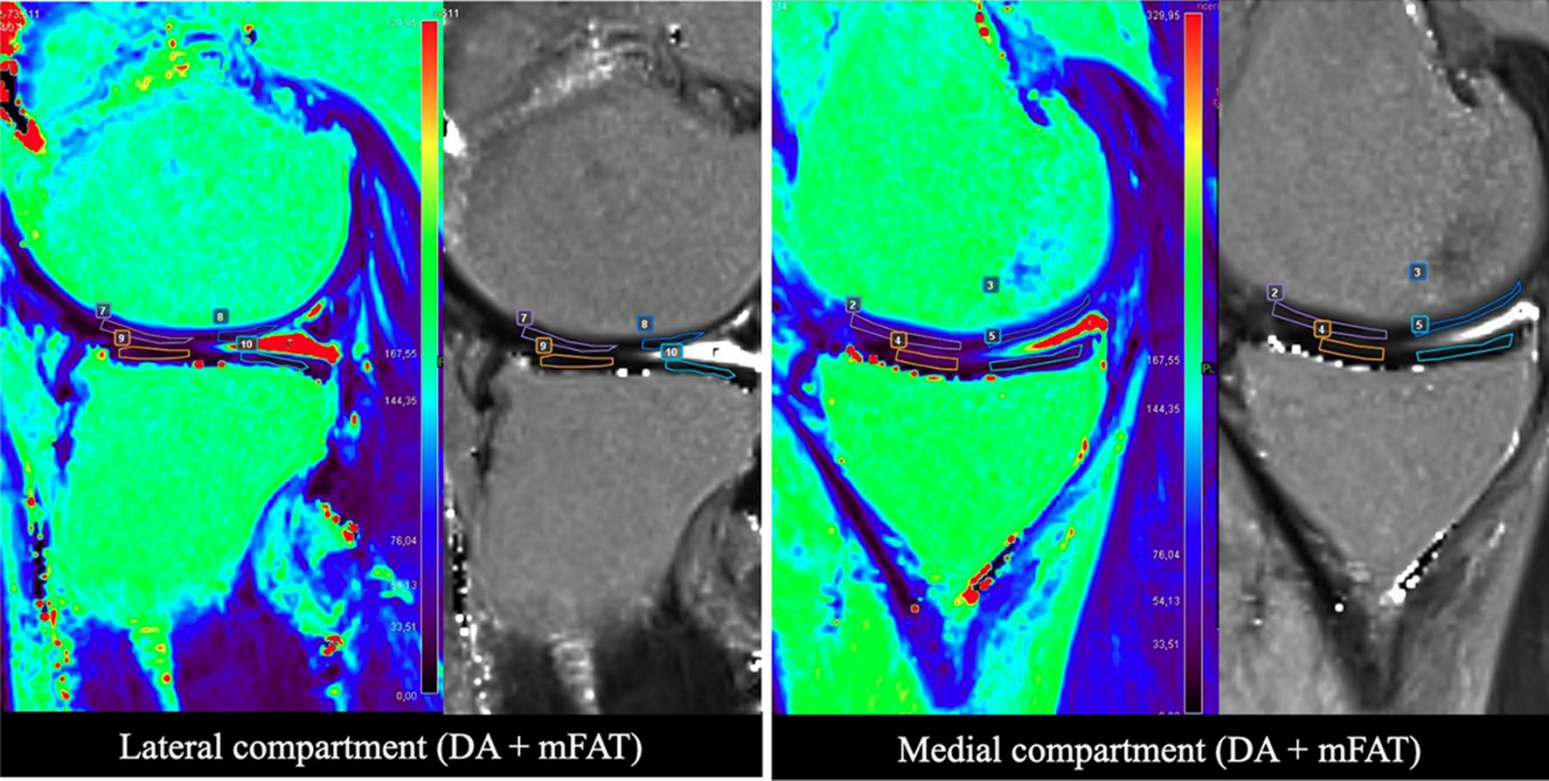
Representative MRI image for patients in AD + mFAT group. T2 mapping evaluation in a 49-year-old male patient treated with AD + mFAT and diagnosed with an MRI Outerbridge grade III at tibio-femoral joint. Regions of interest positioning on both tibia and femur (ROIs) is showed on the lateral side of the knee (left image) and medial side (right image), with corresponding native mapping image used to properly locate the ROIs. T2-mapping values range from 0 to 329.95

**Fig. 5:**
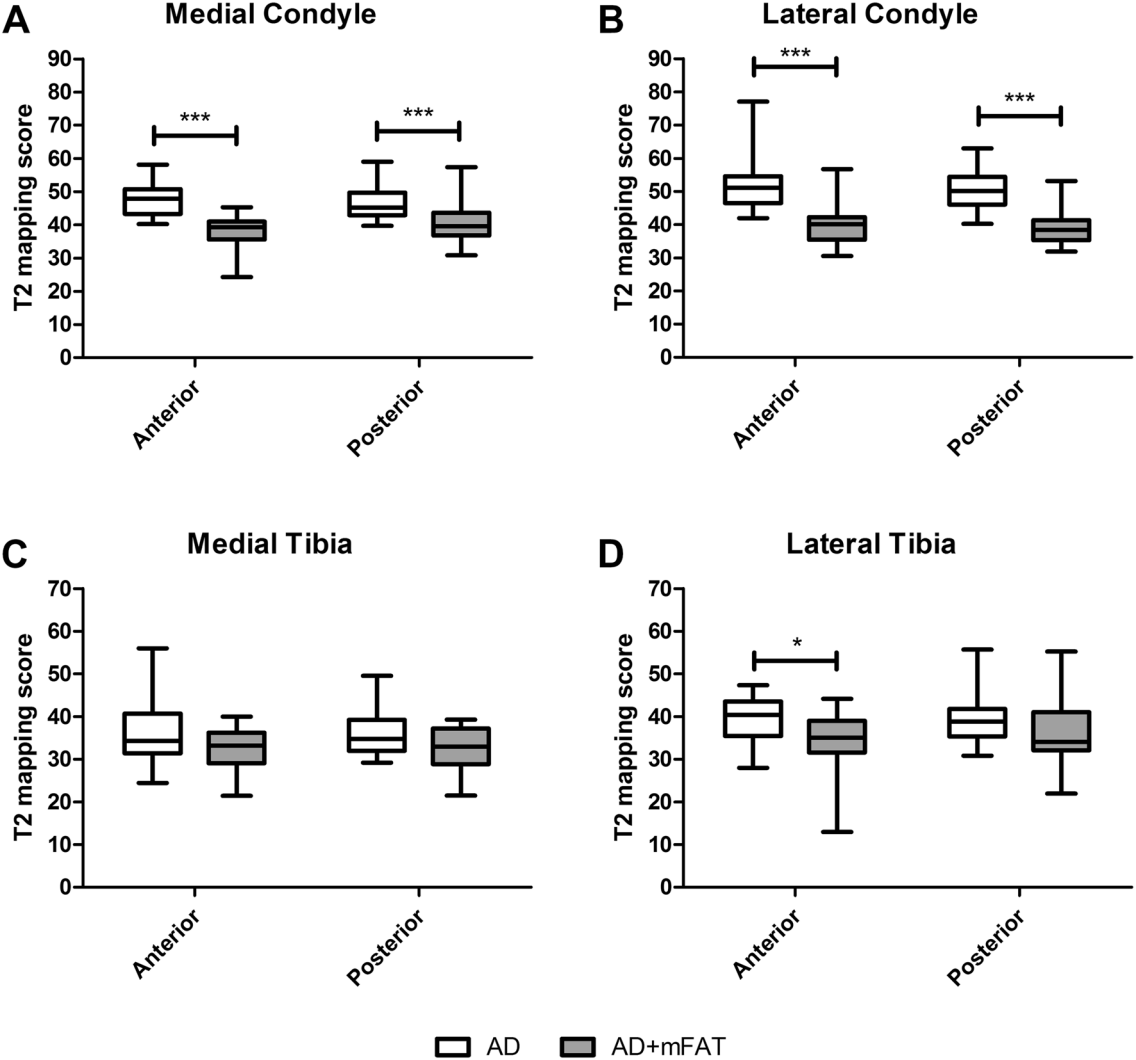
T2 mapping scores. T2 mapping score measured by MRI in the anterior and posterior compartments in AD and AD + mFAT-treated patients. ^*^p < 0.05, ^***^p < 0.001

MRI evaluations were performed at 6-month follow-up. Representative images are reported in Figs. 3 and 4. Significant differences were observed between AD and AD + mFAT-treated patients in terms of T2 mapping score, in anterior/posterior medial condyle and in anterior/posterior lateral condyle compartments (*p* < 0.001, Fig. 5A, B). In all cases, lower values were observed in the AD + mFAT group compared to AD-treated patients, with medians > 30.

No significant differences were found concerning anterior or posterior medial tibia, while in the anterior lateral tibia compartment lower values were observed in AD + mFAT group compared to AD (Fig. 5C, D). Supplementary Table 2 reports median and range for all compartments.

## Discussion

The main finding of this prospective, controlled, randomized study is that the injection of microfragmented adipose tissue in the arthroscopic setting improved functional outcomes in patients affected by knee OA.

Indeed, the use of adipose tissue derivatives produced at the point of care represents a promising strategy for the treatment of OA symptoms [20]. A large number of case series demonstrated the effectiveness of these products when used as an injective treatment alone up to 12 and 24 months [1]. The idea of using mFAT in association with surgical procedures would represent a further step to exploit the regenerative potential of this product to enhance the outcomes of the treatment. Few reports up to today demonstrate that patients would benefit from surgeries (mainly osteotomy and AD) augmented with mFAT injection, and that the procedure is characterized by a high safety profile [5, 21, 33, 34, 36]. This study confirmed the safety of the procedure, with a single procedure-related adverse event (a small hematoma at the harvesting site of the adipose tissue, in the thigh), which resolved spontaneously in few days. To the best of the authors’ knowledge, this study represents the first randomized and controlled trial evaluating the effect of mFAT compared to the index surgery alone. According to the meta-analysis by O’Connor and colleagues published in 2022 [28], arthroscopic debridement is nowadays considered to provide little or no clinically important benefit in the treatment of severe knee OA. It is notable that mFAT was able to foster higher improvements compared to AD alone, reaching minimally clinical important difference (MCID). Indeed, changes in AD + mFAT compared to AD reached MCID for VAS [27] (MCID: 1.0; between-groups difference of 1.1 mm at both 6 months and final follow-up), KOOS-PS [39] (MCID:10; between groups difference of 12.7 and 9.9 points at 6-month and final follow-up, respectively) and KSS [18] (MCID: 9; between-groups difference of 9.9 points at 6-month follow-up) (Supplementary Table 1). Unfortunately, the dropout rate was higher than expected, probably due to the influence of COVID-19 pandemic. This resulted in a reduced power of the statistical tests, especially at the final follow-up (68%). This may explain the lack of significance in the results despite the improvements in AD + mFAT group.

Beside subjective functional improvement, it is also interesting to notice that patients treated with AD + mFAT in this trial showed significantly better T2 mapping MRI results compared to those who underwent non-augmented AD. Indeed, previous reports demonstrated that the intra-articular injection of mFAT was able to improve the glycosaminoglycan (GAG) content of knee cartilage as observed by Delayed Gadolinium-Enhanced MRI of Cartilage (dGEMRIC) at 12 and 24 months from the procedure [4, 12]. Then, the results of this study are compatible with an improvement in cartilage tissue quality possibly due to the enhancement of GAG content.

The evaluation of subjective and objective elements represents a strength of this study. Indeed, even if non-conclusive due to the high inter-individual variability, the testing for blood markers of collagen type II synthesis (PIIINP) [14] and degradation (CTx-II) [9] showed at 6 months a trend for increase in the former and decrease in the latter, suggesting a correlation between this markers and the improvement of patients conditions. Previous reports identified an association between the stage of OA pathology and these markers [14], and in the present study, this seems to be maintained with patients in the AD + mFAT group, who demonstrated worst conditions at baseline, reported consistently reduced PIIINP and increased CTx-II compared to the patients in the AD group.

The main limitation of this work is the lack of blinding of patients and assessors. Indeed, the performance of an additional procedure (liposuction) made it impossible to maintain blinding, since the performance of sham liposuction would have been considered unethical both by Authors of the study and by the IRB. In addition, final follow-up was performed by phone interview, and thus clinician-reported score (KSS) and MRI evaluations were not available at this time-point. The unbalance in the two groups due to chance in the randomization process in terms of baseline scores and conditions represents a further limitation of this study. Suitable statistical methods were employed to balance this difference, but it is possible that this bias influenced the outcomes indirectly, thus not allowing for confounders control. Another limitation is represented by the lack of baseline T2 mapping scores that would have strengthened the significance of the observed improvements in the AD + mFAT group. Nevertheless, the fact that patients in the two groups reported similar profile in terms of Outerbridge classification allows to speculate that the better imaging outcomes of AD + mFAT group are not due to a pre-existing better condition at baseline, but it is the result of the additional treatment. Indeed, all other indexes (PROMs, blood markers) indicate that patients in the AD + mFAT group bear a worse condition at baseline compared to patients treated with AD alone, and thus it is unlikely they presented better conditions at baseline in terms of tissue quality measured by imaging techniques.

## Conclusions

Overall, the results of this study demonstrate that mFAT can be effective in the treatment of knee OA in an arthroscopic setting, in terms of both subjectively reported symptoms and tissue quality observed by MRI T2 mapping at 6 months post-operatively. In addition, benefits of mFAT were still observable at ~ 24-month follow-up, showing overall better results compared to patients treated with AD only. This is the first randomized controlled trial investigating the effect of mFAT combined with a surgical procedure, and it confirms the favorable safety profile of this treatment as well as it supports the rationale of mFAT application in knee OA. Nevertheless, given the unblinded study design adopted, further evidences are needed to draw definitive conclusions.

## Supplementary Information

Below is the link to the electronic supplementary material.

Supplementary file1 (DOCX 18 KB)

Supplementary file2 (DOCX 12 KB)

## Abbreviations

ACL: Anterior cruciate ligament
ANOVA: Analysis of variance
BMI: Body Mass Index
CTx-II: Cross-linked C-Telopeptide of Type II collagen
AD: Arthroscopic debridement
dGEMRIC: Delayed gadolinium enhanced MRI of cartilage
GAG: Glycosaminoglycan
KL: Kellgren–Lawrence classification
KOOS-PS: Knee Injury and Osteoarthritis Outcome Score—Physical Function Shortform
KSS-F: Knee Society Score—function
KSS: Knee Society Score
LCL: Lateral collateral ligament
mFAT: MicroFragmented Adipose Tissue
MRI: Magnetic resonance imaging
OA: OsteoArthritis
PIIINP: N-Terminal Procollagen III Propeptide
PROMs: Patient-reported outcome measures
PT (INR): Prothrombin Time Test (International Normalized Ratio)
RCT: Randomized controlled trial
ROIs: Regions of interest
SF-12 PCS: Short Form-12 Physical Component score
SF-12 MCS: Short Form-12 Mental Component score
TE: Time of echo
TR: Time of repetition
TSE: Turbo spin-echo
VAS: Visual Analog Scale
WOMAC: Western Ontario and McMaster Universities osteoarthritis index
