# Supplementary material for: Micro-fragmented adipose tissue (mFAT) associated with arthroscopic debridement provides functional improvement in knee osteoarthritis: a randomized controlled trial
Source: Knee Surg Sports Traumatol Arthrosc. 2022 Aug 30;31(8):3079–90. doi: 10.1007/s00167-022-07101-4 (PMC9424810; doi:10.1007/s00167-022-07101-4)
Supplement: Supplementary file 1 — Supplementary file1 (DOCX 18 KB) [file 167_2022_7101_MOESM1_ESM.docx]

Supplementary Table 1. Clinical scores in AD and AD+mFAT groups at baseline and changes at 6- and 24-month follow-ups.

|  | Baseline values  (n=67) | Values at 6 months  (n=67) | Change at 6 months  (n=67) | Values at final follow up  (n=55) | Change at final follow up  (n=55) |
| --- | --- | --- | --- | --- | --- |
| VAS  *AD*  *AD+mFAT* | 7.2±1.0  6.9±1.0 | 3.6 ± 2.7  3.0 ± 2.6 | -3.6±3.1***  -4.0±2.6*** | 4.5 ± 3.0  3.3 ± 2.7 | -2.6±3.4***  -3.7±2.4*** |
| KOOS-PS  *AD*  *AD+mFAT* | 55.1±14.2  49.2±15.5^#^ | 66.8 ± 20.8  73.6 ± 17.6 | +11.7±20.2**  +24.4±22.5***^,#^ | 67.8 ± 15.9  69.1 ± 16.6 | +10.6±16.1*  +20.5±22.2*** |
| KOOS-PS A  *AD*  *AD+mFAT* | 8.9±2.5  9.3±2.5 | 5.81 ± 3.74  4.8 ± 3.8 | -3.1±3.8***  -4.5±4.2*** | 6.41 ± 3.86  6.1 ± 3.8 | -2.5±4.6**  -3.2±4.9** |
| KOOS-PS F  *AD*  *AD+mFAT* | 6.4±3.5  8.2±2.3^#^ | 4.39 ± 4.08  3.2 ± 3.3 | -2.0±3.9**  -5.0±4.6***^,##^ | 3.96 ± 2.94  3.8 ± 3.5 | -2.0±3.5**  -4.7±4.2***^,#^ |
| KSS  *AD*  *AD+mFAT* | 56.5±10.8  50.5±13.9^###^ | 71.4±18.2  75.3 ± 17 | +14.9±15.9***  +24.8±23.5*** | n.a. | n.a. |
| KSS-F  *AD*  *AD+mFAT* | 69.5±19.5  67.8±16.5 | 83.7±18.9  89.7 ± 15.4 | +14.2±19.4***  +21.9±18.8*** | n.a. | n.a. |
| SF-12P  *AD*  *AD+mFAT* | 35.0±7.8  34.8±7.8 | 45.1 ± 10.0  47.3 ± 9.6 | +10.1±10.4***  +12.5±10.8*** | 46.1 ± 9.4  44.1 ± 10.0 | +10.6±10.5***  +9.0±10.7*** |
| SF-12M  *AD*  *AD+mFAT* | 52.2±10.6  47.9±10.8 | 53.9 ± 8.8  52.4 ± 11.5 | +1.8±10.5  +4.6±12.4* | 50.1 ± 8.9  49.7 ± 9.1 | -3.6±12.4  +2.0±10.0 |
| WOMAC  *AD*  *AD+mFAT* | 45.7±19.7  48.1±17.7 | 27.6±20.5  22.3 ± 22.7 | -18.1±19.6***  -25.8±24.0*** | 27.5± 8.8  23.8 ± 19.7 | -15.6±20.0**  -25.1±25.0*** |

^*^p<0.05, ^**^p<0.01, ^***^p<0.001 vs baseline; ^#^p<0.05, ^##^p<0.01, ^###^p<0.001 vs AD group; n.a. =not available.
