# Supplementary material for: Micro-fragmented adipose tissue (mFAT) associated with arthroscopic debridement provides functional improvement in knee osteoarthritis: a randomized controlled trial
Source: Knee Surg Sports Traumatol Arthrosc. 2022 Aug 30;31(8):3079–90. doi: 10.1007/s00167-022-07101-4 (PMC9424810; doi:10.1007/s00167-022-07101-4)
Supplement: Supplementary file 2 — Supplementary file2 (DOCX 12 KB) [file 167_2022_7101_MOESM2_ESM.docx]

Supplementary Table 2. T2 mapping score in different knee compartments.

| Compartment | DA | DA+mFAT |
| --- | --- | --- |
| Anterior Medial Condyle^a^ | 47.9 (40.2-58.1) | 39.3 (24.3-45.2)^###^ |
| Posterior Medial Condyle^a^ | 45.2 (39.7-59.0) | 39.6 (30.9-57.4) ^###^ |
| Anterior Lateral Condyle^a^ | 51.1 (41.9-77.0) | 40.1 (30.6-56.8) ^###^ |
| Posterior Lateral Condyle^a^ | 50.1 (40.2-63.0) | 38.4 (31.9-53.1) ^###^ |
| Anterior Medial Tibia^a^ | 34.4 (24.5-56.1) | 33.2 (21.4-40.0) |
| Posterior Medial Tibia^a^ | 34.8 (29.2-49.5) | 33.0 (21.5-39.3) |
| Anterior Lateral Tibia^a^ | 40.4 (28.0-47.4) | 35.1 (13.0-44.2)^#^ |
| Posterior Lateral Tibia^a^ | 38.8 (30.8-55.7) | 34.1 (21.9-55.3) |

^a^Data are expressed as median (range).
^#^p<0.05, ^###^p<0.001 vs DA group.
